# Antioxidant and Cell-Signaling Functions of Hydrogen Sulfide in the Central Nervous System

**DOI:** 10.1155/2018/1873962

**Published:** 2018-02-04

**Authors:** Ulfuara Shefa, Min-Sik Kim, Na Young Jeong, Junyang Jung

**Affiliations:** ^1^Department of Biomedical Science, Graduate School, Kyung Hee University, 26 Kyungheedae-ro, Dongdaemun-gu, Seoul 02447, Republic of Korea; ^2^Department of Applied Chemistry, College of Applied Sciences, Kyung Hee University, Deogyeong-daero, Giheung-gu, Yongin-si, Gyeonggi-do 17104, Republic of Korea; ^3^Department of Anatomy and Cell Biology, College of Medicine, Dong-A University, 32 Daesingongwon-ro, Seo-gu, Busan 49201, Republic of Korea; ^4^East-West Medical Research Institute, Kyung Hee University, 26 Kyungheedae-ro, Dongdaemun-gu, Seoul 02447, Republic of Korea; ^5^Department of Anatomy and Neurobiology, College of Medicine, Kyung Hee University, 26 Kyungheedae-ro, Dongdaemun-gu, Seoul 02447, Republic of Korea

## Abstract

Hydrogen sulfide (H_2_S), a toxic gaseous molecule, plays a physiological role in regulating homeostasis and cell signaling. H_2_S is produced from cysteine by enzymes, such as cystathionine *β*-synthase (CBS), cystathionine *γ*-lyase (CSE), cysteine aminotransferase (CAT), and 3-mercaptopyruvate sulfurtransferase (3MST). These enzymes regulate the overall production of H_2_S in the body. H_2_S has a cell-signaling function in the CNS and plays important roles in combating oxidative species such as reactive oxygen species (ROS) and reactive nitrogen species (RNS) in the body. H_2_S is crucial for maintaining balanced amounts of antioxidants to protect the body from oxidative stress, and appropriate amounts of H_2_S are required to protect the CNS in particular. The body regulates CBS, 3MST, and CSE levels in the CNS, and higher or lower levels of these enzymes cause various neurodegenerative diseases. This review discusses how H_2_S protects the CNS by acting as an antioxidant that reduces excessive amounts of ROS and RNS. Additionally, H_2_S regulates cell signaling to combat neuroinflammation and protect against central neurodegenerative diseases such as Alzheimer's disease (AD), Parkinson's disease (PD), Huntington's disease (HD), and amyotrophic lateral sclerosis (ALS).

## 1. Introduction

Hydrogen sulfide (H_2_S) is a colorless toxic gas with the characteristic odor of rotten eggs [1]. It is produced by decomposition of organic matters and is found in natural gas, petroleum, and volcanic and sulfur-spring emissions [2] under ambient temperature and pressure. Although H_2_S is toxic, it plays a physiological role in the nervous system [3]. H_2_S functions in the secretion of corticotrophin-releasing hormone from serotonergic neurons [4, 5] and in the relaxation of smooth muscle [6, 7]. Additionally, H_2_S shields neurons and cardiac muscles from oxidative stresses [5, 8–11] and helps to maintain insulin secretion [11–13].

H_2_S is produced endogenously from cysteine via enzymes, such as cystathionine *β*-synthase (CBS), cystathionine *γ*-lyase (CSE), 3-mercaptopyruvate sulfurtransferase (3MST), and cysteine aminotransferase (CAT). CBS, a pyridoxal-5′-phosphate- (PLP-) dependent enzyme, is expressed mostly in the brain, with marked localization in astrocytes and cerebellar Bergmann glia [3, 14]. Additionally, Northern blot analysis indicates that CBS is expressed in the hippocampus, cerebellum, cerebrum, and brainstem [3]. While CBS is the main source of H_2_S in the human brain, CBS and CSE are expressed in the tissues of various organs, such as the kidney and the liver. CSE is the main contributor to H_2_S levels in the thoracic aorta, ileum, portal, vein, and uterus. In addition to generating H_2_S from cysteine, CBS catalyzes the condensation reaction of homocysteine, whereas CSE cannot perform this function. 3MST is expressed mainly in the brain, and most of the H_2_S produced by 3MST is bound in the form of sulfane sulfur, one of the forms through which endogenous H_2_S is stored [15]. Understanding the specific expression patterns of these three enzymes is useful for designing therapeutic drugs.

H_2_S acts as a signaling molecule in the cell signal transduction pathways in the nervous system, the circulation system, and in many other organs. In the central nervous system (CNS), H_2_S is associated with various physiological processes, including neuroprotection [16] and neurotransmission [17]. H_2_S inhalation has a neuroprotective function in a mouse model of Parkinson's disease (PD) [18]. H_2_S protects neurons from apoptosis and degeneration [19] by exerting anti-inflammatory effects and upregulating antioxidant enzymes [16]. H_2_S protects neurons from oxidative stresses by reducing the level of reactive oxygen species (ROS) and the aggregation of lipid peroxidation products. Furthermore, H_2_S inhibits the biological activity of peroxynitrites (ONOO^−^) formed in the reaction of nitric oxide (NO) with superoxide anion [20]. H_2_S functions as an antioxidant by scavenging ROS directly and by reducing glutathione disulfide (GSSG) [21]. Increased levels of ROS are found at inflammation sites. Removal of ROS can occur by supplying homocysteine, and stimulated H_2_S synthesis expedites the antioxidant activity [22]. High levels of H_2_S cause generation of ROS and reactive nitrogen species (RNS), whereas lower amounts of H_2_S react with hydrogen peroxides (H_2_O_2_), ONOO^−^, and oxide ion (O^2−^) [23]. Additionally, H_2_S functions as an antioxidant by attaching to particular agents, such as glutathione (GSH), superoxide dismutase (SOD), N-nitroarginine methyl ester (L-NAME), and vitamin C [24].

In this review, we discuss the antioxidant roles of H_2_S; the production of H_2_S from various enzymes, such as CSE, CBS, and 3MST; the cell signaling role of H_2_S in the CNS; the importance of producing appropriate amounts of H_2_S from enzymes to maintain proper neuronal function in the CNS; how dysregulation of cell signaling in the production of enzymes responsible for maintaining H_2_S levels in the body can lead to central neurodegenerative diseases, such as Alzheimer's disease (AD), PD, Huntington disease (HD), and amyotrophic lateral sclerosis (ALS); and how neuroinflammation and disease conditions can be prevented by reducing oxidative stress conditions via the antioxidant functions of H_2_S.

## 2. H_2_S-Producing Enzymes

### 2.1. H_2_S Production by CBS

**Figure 1:**
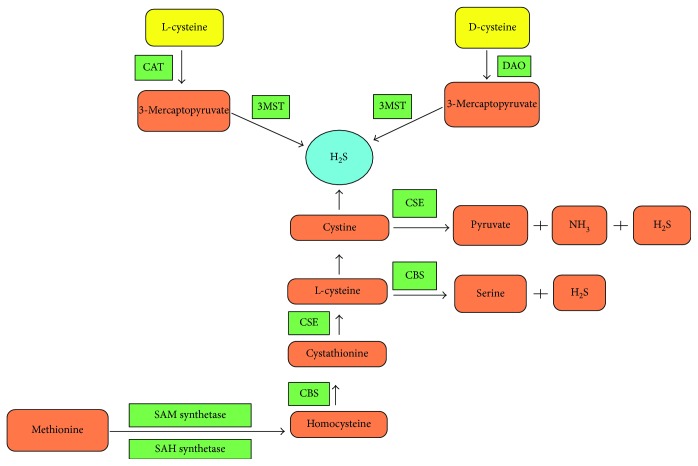
Biosynthesis of hydrogen sulfide (H_2_S) from the cystathionine *β*-synthase (CBS), cystathionine γ-lyase (CSE), cysteine aminotransferase (CAT), and 3-mercaptopyruvate sulfurtransferase (3MST). From the metabolism of methionine, homocysteine is converted to cystathionine and H_2_S is produced from the L-cysteine and homocysteine by enzymes CBS and CSE. L-cysteine in converted to serine and H_2_S with the enzyme CBS. Cystine is converted to pyruvate, ammonia (NH_3_), and H_2_S with enzymes CSE. 3MST works in two ways such as 3MST/CAT and 3MST/DAO (D-amino acid oxidase) pathways to produce H_2_S from L-cysteine and D-cysteine. SAM: S-Adenosylmethionine synthase; SAH: S-Adenosyl homocysteine hydrolase; DAO: D-Amino acid oxidase.

CBS converts serine and homocysteine to generate cystathionine. Additionally, CBS can produce H_2_S from a combination of cysteine and homocysteine. It is unclear whether CBS forms only cystathionine or produces H_2_S from cysteine and homocysteine [25, 26]. CBS has an important role in the regulation of homocysteine levels *in vivo*. Mammalian CBS is regulated by posttranslational modifications and contains a redox-sensitive heme cofactor. The ferrous form of CBS, which forms under local oxidizing conditions, is less active than the ferric form [27]. Carbon monoxide (CO) binds CBS in the ferrous state and inhibits the catalytic activity of CBS [28]. As the redox potential of the Fe^3+^/Fe^2+^ couple in CBS is very low (−350 mV), the availability of CBS entering the ferrous state is controversial. CO reversibly inhibits CBS in the presence of a physiologically relevant reducing system, such as methionine synthase reductase and nicotinamide adenine dinucleotide phosphate (NADPH) [29]; therefore, crosstalk is exhibited between the CO and H_2_S systems. S-Adenosylmethionine (SAM), another metabolite that allosterically activates CBS (Figure 1) [30], is a major methyl donor and the precursor of homocysteine. SAM activates CBS by combining with the carboxy-terminal domain of CBS, which increases H_2_S production [31].

Activation of astrocyte and microglia cell inflammation reduces expression of CBS, leading to diminished levels of H_2_S in the brain. Endogenous H_2_S in the brain is generated mainly by CBS, and altering CBS expression can change the H_2_S levels. Various endogenous and exogenous compounds, such as epidermal growth factor (EGF), which transforms tumor growth factor-*α* (TGF-*α*) and cyclic adenosine monophosphate (cAMP), can increase CBS messenger RNA (mRNA) transcription, which is irregularly maintained in some diseases. These observations suggest several pharmacological targets for combating CNS diseases. CBS expression is threefold higher in patients with Down's syndrome than in normal controls, whereas CBS expression is lower in children with high IQ scores [32]. These findings suggest that overexpression of CBS may have a negative influence on cognitive function. Homocysteinemia is caused by an absence of CBS [32]. In rat hippocampal slices, H_2_S generation from CBS is maintained by calcium (Ca^2+^)/calmodulin and increased by L-glutamate. N-Methyl-D-aspartate (NMDA) and *α*-amino-3-hydroxy-5-methyl-4-isoxazolepropionic acid receptor (AMPA) are inhibited by amino-phosphonopentanoate (AP-5) and 6-cyano-7-nitroquinoxaline-2,3-dione (CNQX), which demonstrates their involvement in this process. The brains of patients with AD show a ~55% reduction in H_2_S levels, whereas L-cysteine levels are unchanged, and CBS activity remains. Chronic H_2_S exposure impairs fetal neuronal development and monoamine neurochemistry in rats. Therefore, H_2_S may have functional involvement in neurodegenerative diseases. CBS can act as an antioxidant inhibitor of peroxynitrite-mediated processes through activation of NMDA receptors. The involvement of the NMDA receptor, with the resulting alteration of long-term potentiation (LTP) in the hippocampus, was the first biological effect described in patients with Down's syndrome who had enhanced concentrations of H_2_S in their cerebral spinal fluid (CSF). This increased concentration may be because CBS is encoded on chromosome 21 and is overexpressed in these patients [33].

CBS is considered the main enzyme in the CNS that produces H_2_S in the brain. H_2_S is produced by CBS by using cysteine and homocysteine as substrates, and various factors, such as the EGF conversion to TGF-*α* and cAMP, are related to producing this enzyme. Understanding the regulation of CBS in the brain may lead to the development of potential therapeutic treatments for many CNS diseases.

### 2.2. H_2_S Production by CSE

CSE converts cysteine to H_2_S, pyruvate, and ammonia; however, this enzyme can also use homocysteine and cysteine as a substrate for H_2_S production (Figure 1) [25, 34]. Rat CSE also uses cysteine (the disulfide form of cystine) as a substrate in H_2_S production [35, 36]. In this case, cysteine persulfide is formed in the presence of a reductant to release H_2_S. The cysteine concentration is extremely low in the reducing environment of cells, but it contributes to H_2_S biogenesis under normal conditions. CSE is thought to be the most prominent enzyme for generating H_2_S in mammalian tissues. CSE-deficient mice show a profound depletion of H_2_S in peripheral tissues [37]. CSE activity has been detected in brain tissue lysates [38], and murine CSE protein expression has been found in imaging studies of the brain [39, 40]. In contrast, CBS protein is expressed mostly in astrocytes [41]. The production of H_2_S from cystine is significantly decreased in brain homogenates from CBS-knockout mice, which demonstrates that CBS is the main source of H_2_S in the brain [42].

CSE, a PLP-dependent enzyme, is found mostly in the liver and kidney and in vascular and nonvascular smooth muscles. CSE exists in the small intestine and stomach of rodents [43]. CSE regulation is less well characterized than that of CBS regulation. Upregulation of CSE is caused by S-nitroso-N-acetylpenicillamine (SNAP), a type of NO donor. Another NO donor, sodium nitroprusside (SNP), enhances CSE activity. Additionally, H_2_S interacts with NO to facilitate NO function in vasorelaxation [6, 44, 45]. CSE-knockout mice deficient in the H_2_S-producing enzyme CSE develop hypertension [37]. Although endogenous production of H_2_S is poorly understood, it plays roles in reducing oxidative stress and in posttranslational protein modification [46–48].

CSE produces H_2_S in the brain and has various physiological roles in maintaining body functions. Similar to CBS, it serves as an important marker of the progression of many CNS diseases.

### 2.3. H_2_S Production by 3MST and CAT

Recent studies have revealed that 3MST and CAT enzymes produce H_2_S from cysteine in the brain [49, 50]. The brains of CBS-knockout mice show the presence of a different H_2_S-producing enzyme [51], and the activity of this enzyme requires mitochondrial and cytosolic components. The required mitochondrial components include 3MST and CAT, which acts as a synaptosome, and the cytosolic components include *α*-ketoglutarate [32]. However, 3MST and CAT show enzymatic activities at pH 7.4, which is comparatively alkaline, and the intermediate of CAT catalysis, 3-mercaptopyruvate (3MP), is an unstable molecule that affects the generation of 3MST. These observations imply that this pathway can generate H_2_S under physiologic conditions (Figure 1) [51]. Aspartate, another substrate for CAT, can associate competitively with CAT to inhibit H_2_S generation. A comparison of these enzymes reveals several differences. CBS is found primarily in astrocytes, whereas 3MST is localized mainly in neurons. 3MST generates bound sulfane sulfur more efficiently than does CBS. 3MST transfers sulfur from H_2_S to bound sulfane sulfur, whereas CBS has low capacity for this activity. 3MST is also localized in the thoracic aorta. The presence of 3MST, CAT, and *α*-ketoglutarate in the endothelium suggests that H_2_S can be generated in the endothelium [32].

3MST and CAT are other enzymes that produce H_2_S from cysteine under physiologic conditions and maintain homeostasis by ensuring the balance of H_2_S in the body.

## 3. Bioactivity of H_2_S

There are two possible mechanisms by which H_2_S is released; it can be released immediately after the production by the enzymes and it can be stored and released in response to a physiologic signal. Two forms of sulfur stores in cells have been identified [52, 53]. Acidic conditions release H_2_S from acid-labile sulfur. Another form of storage, bound sulfane sulfur, releases H_2_S under reducing conditions [54]. Acid-labile sulfur is contained in iron–sulfur complexes that play a pivotal role in a wide range of redox reactions in the respiratory chain of mitochondria. H_2_S is released from acid-labile sulfur at pH 5.4 [55]. As the pH in mitochondria is between 7 and 8, it is likely that acid-labile sulfur does not release H_2_S under normal physiologic conditions.

H_2_S can be associated into proteins as bound sulfane sulfur [56]; thus, enzymatically produced H_2_S may be stored as bound sulfane sulfur. Cells expressing 3MST and CAT have increased levels of bound sulfane sulfur [55] compared to cells expressing a defective mutant of 3MST that does not produce H_2_S [51]. The level of sulfur is intracellularly dependent on the H_2_S-generating activity of 3MST; H_2_S generated by 3MST is preserved as bound sulfane sulfur in cells. In the presence of major cellular reducing substances GSH and cysteine at their physiologic concentrations, H_2_S is released from lysates of cultured neurons and astrocytes at pH 8.4 [55]. Because the reducing activity of thiols is higher under alkaline conditions than at a neutral pH, H_2_S release can be detected at pH values higher than 8.4. Although systemic pH changes of up to approximately 0.2 constitute either alkalosis or acidosis, it is possible that the pH can be altered to a greater extent locally. As neurons are excited, sodium ions (Na^+^) enter and potassium ions (K^+^) exit from cells, which results in high potassium concentrations in the extracellular environment. This depolarizes the membrane of the surrounding astrocytes and activates their Na^+^/bicarbonate (HCO_3_^−^) cotransporters. The entrance of HCO_3_^−^ causes alkalization of the cells. The newly produced H_2_S stays in equilibrium with its anionic form bisulfide (HS^−^), with an intracellular ratio of H_2_S to HS^−^ of 1 : 4. However, it is unclear whether HS^−^ anion and free H_2_S contribute equally to cell signaling. H_2_S has the ability to traverse cell membranes without the need of a facilitator [57], whereas it was previously thought that HS^−^ anions are not able to cross cell membranes and, hence, could target only intracellular proteins. In contrast, a channel permeable to HS^−^ anions was recently found in the bacterium *Clostridium difficile*, demonstrating that the signaling role of HS^−^ anions may be confined to HS^−^ anion-producing cells [58].

Signaling of H_2_S is maintained by rapid clearance of H_2_S by various biochemical pathways that metabolize H_2_S. A high rate of H_2_S generation is maintained by its degradation via oxygen-dependent catabolic processes in mitochondria in murine tissues [59]. Additionally, H_2_S can be present in a bound form sulfane, which releases H_2_S in the presence of a reducing agent under alkaline conditions; however, there is no evidence for a physiological function of sulfane sulfur in cellular signaling. H_2_S also reacts with hemeproteins, such as hemoglobin, neuroglia, and cytochrome c oxidase, which may act as links for this gasotransmitter [59]. Also, H_2_S can be methylated in the cytosol by thiol-S-methyl transferase to produce methane thiol, which can be further methylated to become the less toxic compound dimethyl sulfide [60].

After the production from various enzymes, H_2_S stays in equilibrium by forming anions in the body, and further generation of H_2_S is dependent on the degradation rate needed to maintain the physiological functions of the body.

## 4. Detection and Measurement of H_2_S

Intracellular H_2_S levels can be detected and quantified using several methods that have varying levels of sensitivity [61]. H_2_S generation from cysteine or homocysteine using very high substrate concentrations leads to inaccurate detection of H_2_S levels. The most common method for accurate detection of H_2_S levels involves H_2_S trapping with zinc or lead, followed by acidification and reaction with N,N-dimethyl-P-phenylenediamine (DMPD) to produce methylene blue, which can be detected by colorimetry. This process is preferred under acidic conditions and results in the release of bound H_2_S from stored sources. However, this method does not differentiate between free and bound H_2_S. Moreover, this method lacks sensitivity and cannot detect nanomolar amounts of H_2_S. In contrast, gas chromatography can detect H_2_S levels in the nanomolar range and can distinguish between free sulfide and acid-labile sulfide [62]. Measurement of H_2_S in real time [63] is not easily possible; amperometry does allow for monitoring and direct measurement of H_2_S production in real time, but the detection electrodes require frequent calibration, which is accompanied by difficulties related to handling small volumes. Lastly, H_2_S-specified probes can detect local H_2_O_2_ generation in live cells, but these probes are sometimes inadequate for the identification of H_2_S in the submicromolar range [64].

In conclusion, H_2_S can be detected using various methods, such as colorimetry or gas chromatography, but these methods cannot detect or measure H_2_S at the nanomolar or submicromolar range. Although these methods are limited, they can be optimized to detect H_2_S within such limitations, whereas amperometry measures H_2_S in real time.

## 5. Signaling Mechanisms

### 5.1. H_2_S as Signaling Molecules in the CNS

Olas et al. experimentally demonstrated that H_2_S serves a neuroprotective function, maintaining the intracellular pH in microglial cells and limiting the damage to activated microglia at the site of injury. H_2_S inhibits cytochrome c oxidase or causes excessive NMDA receptor stimulation through the secondary transmitter cAMP. NMDA receptors are built from three subunits, NMDAR1, NMDAR2A, and NMDAR2B. Endogenous ligands of the receptor include acid, NMDA, and glutamic acid. After joining the glutamate receptor subunit, phosphorylation occurs inside the NMDAR1 ion channel via protein kinase A (PKA) activity, which is dependent on cAMP [65]. For this reason, the channel opens, and an influx of Ca^2+^ ions is observed. In the next step, the signaling pathway involves changes in the long-term strengthening of synapses, which enhances the efficiency with which nerve impulses travel across synapses. H_2_S affects the function of the hypothalamic–pituitary–adrenal glands [66]. H_2_S decreases release of potassium hormones stimulated by the hypothalamus by acting as a negative regulator of the hypothalamic–pituitary–adrenal glands. This compound also affects intracellular stores of Ca^2+^, stimulating their release inside cells, which causes nerve excitation. It has been demonstrated that H_2_S reduces the cysteine disulfide bond of the NMDA receptor to increase its activity [67]. Eventually, H_2_S-derived polysulfide (H_2_S_n_) increases the activity by producing bound sulfane sulfur in the cysteine residues of the receptors. H_2_S_n_ also activates the channels in astrocytes to enhance intracellular concentrations of Ca^2+^ that facilitate the release of serine, which in turn increases the activity of NMDA receptors. Wang et al. demonstrated the involvement of H_2_S in neuronal cell differentiation [68].

It has been reported that concentrations of H_2_S 10 to 130 *μ*M in the CNS not only activate the NMDA receptor-mediated response but also increase the speed with which LTP occurs [3]. At higher concentrations (320 and 640 *μ*M), sodium hydrosulfide (NaHS) inhibits synaptic transmission. In fact, H_2_S concentrations from 30 to 400 *μ*M produce the opposite effects on neuronal transmembrane potentials in toxicological studies [69]. Expression of gamma aminobutyric (GABA_B_) receptor subunits 1 and 2 is upregulated by H_2_S, whereas expression of the GABA_B_ receptor subunits 2 and 1 is inhibited by hydroxylamine, a nonspecific inhibitor of H_2_S biogenesis [70]. H_2_S affects the levels of epinephrine, norepinephrine, and serotonin in the brain [71]. Additionally, H_2_S enhances intracellular Ca^2+^ in neurons, astrocytes, and microglia by upregulating the influx of Ca^2+^ into the cytoplasm from extracellular and intracellular compartments [72]; this affects the interactions among these cells. Indeed, activation of voltage-dependent Ca^2+^ channels or of transient receptor potential channels by H_2_S is thought to underpin the intracellular increase in Ca^2+^ [44].

To conclude, H_2_S performs a cell-signaling function in the CNS by activating NMDA receptors and increasing intracellular Ca^2+^ by activating voltage-gated sodium channels in neuronal cells. By doing so, it performs antioxidant functions by upregulating generation of GSH and mitigating oxidative stresses in cells.

### 5.2. Potential Molecular Targets in H_2_S Signaling in the CNS

H_2_S has recently been understood to act as a signaling molecule in the CNS. Indeed, H_2_S is involved in the regulation of the pathways and molecules detailed in the following subsections.

#### 5.2.1. cAMP/PKA Signaling Pathway

**Figure 2:**
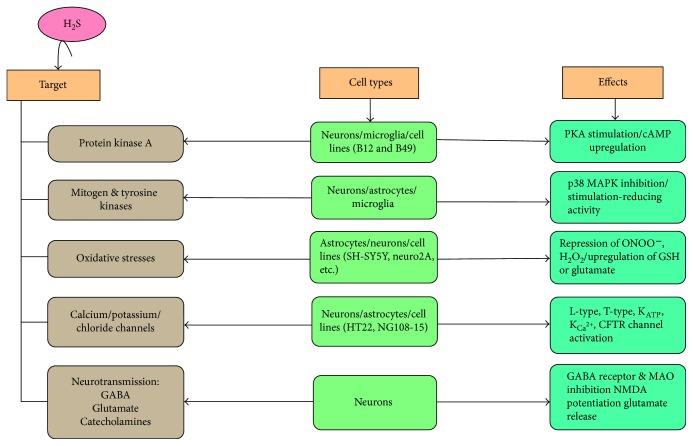
Potential molecular targets in hydrogen sulfide (H_2_S) signaling in the central nervous system (CNS). H_2_S targets protein kinase A (PKA) and activates PKA stimulation or cyclic adenosine monophosphate (cAMP) upregulation which has effects on neurons, microglia, and the cell lines such as B12 and B49. It also activates mitogen and tyrosine kinases which initiates p38 mitogen-activated protein kinase (MAPK) inhibition as well as stimulation of the reducing activity in neurons, astrocytes, and microglia. Oxidative stress has activity on suppression of peroxynitrites (ONOO^−^), hydrogen peroxide (H_2_O_2_), and upregulation of glutathione (GSH) or glutamate. Additionally, H_2_S activates on calcium (Ca^2+^), potassium (K^+^), and chloride (Cl^−^) channels in neurons, astrocytes, and cell lines such as HT22 and NG108–15. Moreover, H_2_S has effects on neurons in neurotransmission such as gamma-aminobutyric acid (GABA) receptor inhibition, N-methyl-D-aspartic acid (NMDA) potentiation glutamate release, and monoamine oxidase (MAO) inhibition. In these ways, H_2_S stimulates molecular targets on the CNS to impart their different functions.

Generation of cAMP by adenylyl cyclase (AC) stimulates PKA, which, in turn, phosphorylates various intracellular proteins; hence, it is involved in the maintenance of brain functions. LTP is produced rapidly by high-frequency presynaptic activation that strengthens the postsynaptic response, continuing presynaptic stimulation. Regulation of LTP requires activation of PKA, which may phosphorylate NMDA receptors and enhance Ca^2+^ permeability, facilitating both the early and late phases of LTP (Figure 2) [73]. It has been demonstrated that NaHS, which is a H_2_S donor, enhances cAMP generation in primary cultures of the cerebral cortex, cerebellum neurons, and glial cells in a concentration-dependent manner [74]. These studies demonstrated that H_2_S may modulate the activity of NMDA receptors through changing intracellular cAMP levels and upregulating the induction of LTP. Activation of the cAMP/PKA pathway also stimulates ryanodine receptors in the brain, leading to calcium-induced calcium release [73].

#### 5.2.2. Tyrosine and Mitogen Kinases

Tyrosine kinase (RTK) receptors are regarded as a part of a large family of cell surface receptors with intrinsic RTK activity [11]. The possibility that H_2_S may upregulate the reducing activity and protect neurons against oxidative stress acquired through activation of upstream RTK (Figure 2). It is likely that H_2_S stimulates epidermal growth factor receptor (EGFR) type RTK, as experiments with tryphostin A23 inhibited the effect of H_2_S or WST-8, a tetrazolium salt, compared with a control analogue trypostin A1 that lacked EGFR inhibitory activity [11]. The activation of EGFR by H_2_S is consistent with observations that H_2_S promotes NMDA signaling and LTP, which are similar to the effects observed with EGF [73]. Mitogen-activated protein kinases (MAPKs) are a large family of kinases divided into five distinct groups in mammals; they are activated by external stimuli, and their activation stimulates downstream effectors through phosphorylation. MAPKs maintain many cellular activities, including apoptosis, differentiation, metabolism, mobility, cell division, and survival [75]. It has been demonstrated recently that H_2_S inhibits LPS-imparted NO production in microglia through inhibition of p38 MAPK. This indicates that H_2_S may be useful in the neuroprotection involved in the treatment of cerebral ischemia and neuroinflammatory diseases [76].

#### 5.2.3. GSH and Oxidative Stress

It has been noted that H_2_S inhibits peroxynitrite-imparted cytotoxicity, intracellular protein nitration, and protein oxidation in human neuroblastoma SH-SY5Y cells. These studies demonstrate that H_2_S has the potential role to act as an inhibitor of peroxynitrite-mediated processes *in vivo* and reflect the potential antioxidant action of H_2_S [77]. Hence, H_2_S protects against the activity of peroxynitrite-mediated processes *in vivo*, which suggests the potential antioxidant action of H_2_S. Hence, H_2_S protects against the activity of peroxynitrite in SH-SY5Y neuroblastoma cells, assembly via enhanced GSH production (Figure 2). In the same way, in HT22 neuronal cells and primary cultured immature cortical neurons, H_2_S inhibits cell toxicity related to apoptosis, a form of oxidative glutamate toxicity that operates independently from the glutamatergic signaling at ionotropic glutamate receptors [11].

#### 5.2.4. Effects of H_2_S on Ca^2+^, Potassium (K^+^), and Chloride (Cl^−^) Channels in the CNS

In neurons, physiological concentrations of H_2_S generate a biphasic response in dorsal raphe seronergic neurons; this response is characterized by initial, rapid-onset depolarization followed by sustained hyperpolarization. The primary depolarization response is sensitive to inhibition via removal of external Ca^2+^ or blockage using cadmium but not tetradotoxin, which is a sodium channel blocker; this highlights the participation of extracellular Ca^2+^ influx in the initial depolarization response [69]. Plasma membrane voltage-gated channels that may be activated by H_2_S include L-type channels and T-type Ca^2+^ channels. L-type Ca^2+^ channels are coded by four different genes in mammals, Cav1.1–Cav1.4. L-type channels are expressed in neurons and endocrine cells and regulate many processes, such as neurohormones and neurotransmitter secretion, gene expression, mRNA stabilization, neuronal survival, synaptic efficiency, and the activity of other ion channels, such as NMDA receptors [78]. Neurons express Cav1.2 and Cav1.3 subtypes. The subtypes differ in their activation thresholds, sensitivity to dehydropyridine antagonists, activation kinetics, and subcellular distributions. H_2_S has led to neuronal death and an increase in [Ca^2+^]i in rat cerebellar granule neurons, but both of these outcomes have been blocked by L-type channel-specific blocker, nifedipine or nimodipine, demonstrating that H_2_S acts on L-type Ca^2+^ channels. In astrocytes, Ca^2+^ waves induced by H_2_S were found to be blocked by nifedipine [79].

Using particular K^+^ channel blockers, gliclazide and apamin, respectively, researcher has found that physiological concentrations of H_2_S activate both adenosine triphosphate- (ATP-) sensitive potassium and calcium (K_ATP_ and K_Ca^2+^_) channels in the hypothalamus and dorsal raphe serotonergic neurons (Figure 2) [80]. H_2_S was also found to stimulate K_ATP_ channels in neuronal cell lines. Blockade of K_ATP_ channels with the blockers glibenclamide and glipizide counteracted the survivability imparted by H_2_S during oxytocic insult; this finding was confirmed using the K_ATP_ activator pinacidal [9]. K_ATP_ channels also play roles in seizure control, mediating neurotransmitter release from presynaptic neurons, and mediating neuroprotection during hypoxic challenge; hence, it is tempting to speculate that H_2_S plays a neuroprotective role through activation of K^+^ channels [81]. Additionally, H_2_S has been found to activate cystic fibrosis transmembrane conductance regulator (CFTR) Cl^−^ channels in HT22 neuronal cell lines, leading to neuroprotection during oxytosis (Figure 2). This was observed via dose-dependent repression of neuroprotection due to H_2_S using specific CFTR blockers, 5-nitro-2-(3-phenylpropylamino) benzoic acid (NPPB) and indolylacetic acid (IAA), and was confirmed using the CFTR activator levamisole [9]. Taken together with our recent observation of H_2_S stimulating Cl^−^/HCO3^−^ transporters in smooth muscle cells, these studies suggest that H_2_S in the CNS is involved in the regulation of inhibitory K^+^ channels and therefore plays a pivotal role in mediating excitability [82].

#### 5.2.5. Effect of H_2_S on GABA-Mediated, Glutamate-Mediated, and Catecholaminergic Neurotransmission

GABA is the major inhibitory transmitter within the mammalian CNS: 20–30% of all synapses in the CNS employ GABA as their transmitter [83]. GABA-mediated inhibition in the CNS is critical, as loss of GABAergic inhibition leads to seizures and neuronal hyperexcitability. There are three types of receptors for GABA in the CNS: GABA_A_, GABA_B_, and GABA_C_ receptors; these produce slow, prolonged inhibitory signals that modulate the release of neurotransmitters [84]. H_2_S has been found to promote amelioration of hippocampal damage caused by recurrent febrile seizures via a reversal of the loss of the GABABR1 and GABAB2 caused by the seizures [85]. This amelioration was traced to the elevated mRNA and protein levels of these GABA receptors, possibly due to acute (H_2_S-induced) increases in [Ca^2+^]i, following Ca^2+^-dependent transcription [85]. This may affect the excitation or inhibition balance that is perturbed during fever by affecting slow, accelerated inhibitory signals and neurotransmitter release. It is possible that H_2_S accelerates inhibitory signals on transmitter release and may have potential uses in the treatment of excitatory diseases, such as epilepsy [70].

Although there is no direct evidence of H_2_S agonist activity on NMDA receptors, accumulating evidence suggest that H_2_S may generate physiological or pathological functions through maintaining NMDA receptors [73]. H_2_S stimulates LTP through potentiation of NMDA receptors. This effect is achieved mainly by H_2_S-imparted activation of the cAMP/PKA pathway [3]. Excessive activation of NMDA causes calcium overload in cells, leading to cell death [86]. Hence, NMDA receptors play essential roles in certain conditions, such as stroke, neuropathic pain, PD, and so forth. NMDA receptor blockers have been found to inhibit H_2_S-imparted cell death in neurons and decrease infarct volume in an *in vivo* rat stroke model [73], demonstrating that H_2_S may impart cell death by opening NMDA receptors (Figure 2). In brief, H_2_S-imparted NMDA signaling may promote excitation and contribute to whether neurons survive or die [87]. Sublethal or lethal concentrations of H_2_S have been reported to inhibit monoamine oxide, leading to an increase in noradrenaline and adrenaline in the hippocampus, striatum, and brainstem but not in the cortex or cerebellum. Because of the myriad effects elicited by catecholamines or adrenoceptors in the CNS, further study is needed to elucidate the importance of the toxicological effects of H_2_S [88].

However, H_2_S activates different receptors and molecular targets as mentioned above paragraphs either individually or in combination to impart neuroprotective effects in the CNS.

## 6. Roles of GSH and H_2_S as Antioxidants in the CNS

GSH is a nonprotein thiol that is present in millimolar amounts in mammalian cells. It is considered less able to potentiate oxidation than cysteine and is good for regulating intracellular redox potential. The essential function of GSH includes its antioxidant activity [89], particularly its function in regulating protein thiol homeostasis and serving as the reaction partner for the detoxification of xenobiotics [90], as a cofactor in isomerization reactions, and in storage and transport from cysteine [91]. In the brain, GSH is an essential antioxidant that is regarded as highly sensitive to perturbation of the equilibrium between the antioxidant system and ROS. Oxidant species are associated with the pathogenesis and advancement of various neurodegenerative diseases, the regulation of redox status, and the antioxidant capacity of the CNS in the period of oxidative stress, which is essential for neuroprotection [92]. Glutamyl cysteine synthatase (GCS) is regulated physiologically either by competitive nonallosteric inhibition by GSH [93] or by the availability of its precursor amino acids. The availability of cysteine is essential for GSH synthesis. Cysteine is produced via the transsulfuration pathway, whereas dietary methionine is transformed to cysteine. Activation of ATP-dependent methionine promotes the generation of SAM and the gradual demethylation and removal of the adenosyl moiety-generated homocysteine. Homocysteine accumulates with serine to produce CBS. The terminal enzyme of the transsulfuration pathway is CSE, which is a PLP-dependent enzyme. It catalyzes the transition of L-cystathionine into L-cysteine; *α*-ketobutyrate and ammonia are the rate-limiting enzymes for the synthesis of cysteine from methionine. Hence, cystine levels in cells may also be increased by transport of cysteine via specialized transporter systems [94]. The significance of the transformation pathway involved in producing cystine for GSH production in the liver is well recognized because any disturbance of this pathway reduces levels of cellular GSH [95].

Moreover, H_2_S has the ability to protect neurons from oxidative stress by enhancing levels of GSH. When extracellular concentrations of glutamate are enhanced, a process known as oxidative glutamate toxicity, the import of cysteine in exchange for glutamate by the cysteine/glutamate antiporter is reduced. Because cysteine is converted to cystine in cells for the production of GSH, a reduction in cystine causes a reduction in the production of GSH. H_2_S conserves cells under conditions of oxidative stress by two mechanisms, by increasing the generation of GSH, by increasing levels of cysteine/cystine transporters, and by redistributing the localization of GSH to mitochondria. As H_2_S is regarded as a reducing substance and as cystine is present in plasma and blood at certain concentrations, H_2_S may inhibit the process by which cysteine is reduced to cystine in the extracellular space and may enhance the transmembrane transport of cysteine into cells for GSH generation. Enhanced cysteine transport contributes to increased production of GSH. Enhanced GSH generation by H_2_S is important under conditions of oxidative stress caused by glutamate. H_2_S enhances both the generation of GSH and its redistribution to mitochondria. Additionally, its generation in mitochondria may occur in the context of reducing oxidative stress [96]. To achieve the protective effect of H_2_S, one should test not only for glutamate toxicity but also for other markers of oxidative stress. In cerebral tissues, glutamate is not entirely liable for causing neuronal damage. The results of H_2_O_2_-imparted oxidative stress should not be ignored. H_2_S retrieves GSH levels which is oppressed by H_2_O_2_, demonstrating that H_2_S conserves cells from various oxidative stress stimuli. H_2_S can also be restored [96]. In the embryonic brain, GSH levels that have been reduced by ischemia reperfusion and cysteine import are further oppressed by glutamate. In brief, H_2_S enhances GSH concentration by intracellularly upregulating the transport of cysteine to a greater extent than it upregulates that of cystine. Additionally, H_2_S enhances the redistribution of GSH into mitochondria. Hence, H_2_S generated in mitochondria plays a role in the conservation of cells under conditions of oxidative stress [49].

Furthermore, although antioxidant activity is produced through a direct interaction between H_2_S and ROS [32], it seems unlikely to be a quantitively efficient mechanism because of the low concentrations of H_2_S compared with those of other antioxidants, such as GSH. Intraperitoneal NaHS treatment of pregnant rats protects the fetal brain from damage caused by ischemia reperfusion, which is compensated for by GSH levels [49]. Supplementation with cysteine facilitates the proliferation and differentiation of neuronal stem cells to neurons and astroglia, which is attenuated by knockdown of CBS expression using small interfering RNA (SiRNA) [68]. The neuroprotective effects of H_2_S can be imparted by its anti-inflammatory and antiapoptosis activities [15] and its stabilization of membrane potentials [97]. Overall, GSH is a pivotal enzyme that reduces oxidative species in the CNS and maintains the H_2_S balance to avoid neurodegenerative conditions in our body.

## 7. Antioxidant Effects of H_2_S in CNS Neurodegenerative Diseases

**Figure 3:**
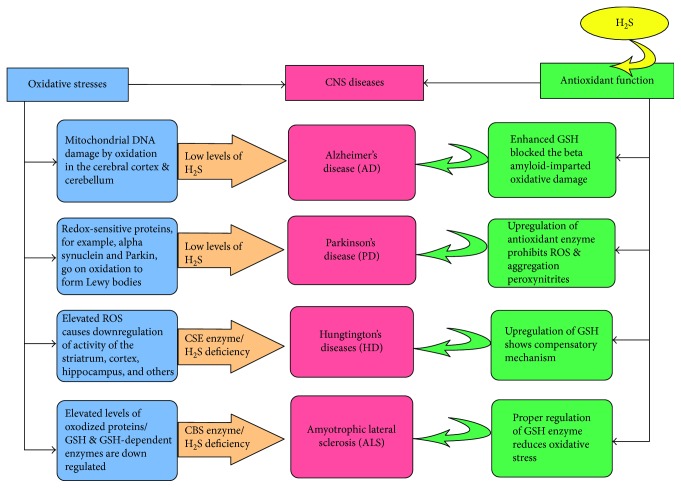
Role of hydrogen sulfide (H_2_S) as antioxidant in the neurodegenerative diseases Alzheimer's disease (AD), Parkinson's disease (PD), Huntington's disease (HD), and amyotrophic lateral sclerosis (ALS). In AD, mitochondrial damage is caused by low levels of H_2_S in the cerebral cortex and cerebellum whereas H_2_S acts as antioxidant by GSH and amyloid beta-mediated oxidative damage. In PD, redox-sensitive proteins such as *α*-synuclein, parkin, and so on form Lewy bodies because of low levels of H_2_S whereas H_2_S imparts antioxidant functions by upregulation of antioxidant enzymes which inhibits reactive oxygen species (ROS) as well as lipid peroxidation products. In HD, elevated levels of ROS causes downregulation of activity in the striatum, hippocampus, and so on because of low supply of H_2_S whereas H_2_S acts as an antioxidant by reducing the excessive ROS. Lastly, in ALS, excessive amounts of proteins are downregulated because of low levels of H_2_S and H_2_S acts as antioxidant by proper regulation of antioxidant enzymes in ALS. In this ways, H_2_S imparts antioxidant functions by modulating oxidative stress conditions in the neurodegenerative diseases.

Several major factors can cause the initiation and progression of neurodegenerative diseases, including oxidative stress, protein misfolding, and protein aggregation. Dysregulation of GSH homeostasis and deactivation of GSH-dependent enzymes are thought to play essential roles in the initiation and advancement of neurodegenerative diseases such as AD, PD, HD, and ALS (Figure 3) [98].

### 7.1. PD

PD is a neurodegenerative disorder affecting more than four million people all over the world. The brain of PD patient is characterized with loss of dopamine-secreting neurons in an area of the midbrain which is known as the substantia nigra (SN), subsequently causing bradykinesia, postural instability, resting tremor, and rigidity of patients [99]. In PD, H_2_S metabolism may be involved. In a mouse model of PD, H_2_S levels in the SN and striatum were lower in control mice [100]. In one experiment, H_2_S introduced through injection or inhalation [18] prevented PD-like abnormalities, including movement dysfunction and microglial activation, from occurring. ROS is associated with the progression of PD; it is thought that impairment of the protective functions of GSH and related enzymes is involved in PD initiation and progression. As an example, postmortem brain tissue from PD patients' samples contained decreased amounts of GSH compared to controls [101]. In PD, many kinds of proteins are associated with cysteine residues, which are sensitive to oxidation. Hence, redox-sensitive proteins such as *α*-synuclein, parkin, and DJ-1 are involved in familial PD. *α*-Synuclein was the first gene found to be involved in familial PD and accumulation of Lewy bodies and subsequent neuronal cell death (Figure 3) [102]. GSSG regulation facilitates this accumulation, and neuronal cell death involved with *α*-synuclein in *Drosophila* can be rescued by interventions that enhances GSH [103].

Some recent studies have found that H_2_S shields neurons against oxidative stress. These studies also found that H_2_S has anti-inflammatory effects on brain cells in PD animal models [104]. The current clinical treatment for PD is levodopa (L-DOPA) replacement therapy to improve symptoms; however, this treatment can result in side effects such as dyskinesia and cannot prevent the advancement of PD. Based on previous studies, plasma homocysteine levels in PD are elevated when patients are treated with L-DOPA [105]. Moreover, current studies indicate that treatment with NaHS can significantly reduce the loss of SN neurons and slow the advancement of motor dysfunction in 6-hydroxydopamine hydrobromide-imparted and rotenone-induced PD models [106]. Additionally, inhalation of H_2_S hinders the movement disorder resulting from 1-methyl-4-phenyl-1,2,3,6-tetrahydropyridine-imparted PD. Hence, H_2_S is thought to provide new ideas for the pathogenesis and clinical treatment of PD [18]. H_2_S plays a role in PD to combat oxidative stresses, combining with the enzyme GSH that acts as an antioxidant.

### 7.2. AD

AD is a catastrophic as well as progressive neurodegenerative disorder featured by extracellular accumulation of amyloid beta (A*β*) protein as well as intraneuronal neurofibrillary tangles (NFTs). The deleterious microglial activation in AD has been supported by analysis of postmortem brains of patients with AD where microglial overactivation occurred before neuronal damage demonstrating a crucial role in the advancement of AD [107]. Oxidative stress is also associated with AD progression. Dissection of postmortem AD brains has shown increased oxidative damage to nuclear and mitochondrial DNA in the cerebral cortex and cerebellum compared to age-matched controls (Figure 3) [108]. In addition, A*β* is thought to be a prooxidant itself, and this characteristic is considered partly liable for ROS production [109]. This kind of oxidation functions in neuronal death, leading to advancement of AD [110]; mutations in GSH-dependent enzymes are reported to increase the risk of AD. For example, a polymorphism in the glutathione peroxidase 1 (GPX1) gene has been identified as a possible risk factor in AD advancement [111]. Increasing intracellular levels of GSH is one defensive approach against AD advancement. N-Acetylcysteine (NAC) can act as a precursor for de novo synthesis of GSH. Mice treated with NAC prior to intracerebroventricular injection of A*β* demonstrated an enhanced ability to learn and increased memory function compared to controls [112]; GSH content is enhanced after NAC treatment. In addition, lipid protein and oxidation are reduced [112]. The enhanced GSH may block the prooxidant effects of the A*β* and prevent onset of the AD-like syndrome or may support more efficient repair of A*β*-imparted oxidative damage.

H_2_S levels are lower in the brains of AD patients than in age-matched healthy people, although expression levels of CBS do not differ between the two groups [113]. Although AD is regarded as a result of decreased production of H_2_S, there may be an associated decrease in neuronal cytoprotection that enhances the harmful effects of damage and neuroinflammation induced by A*β* and oxidative stress [114–116]. Whether the low levels of H_2_S seen in the brain in AD are a cause or a consequence of the disorder is not clear. In an experiment with rats examining whether vascular ischemia was associated with a decrease in viable neuron numbers in the hippocampus, injection with NaHS intraperitoneally markedly protected against neuronal injury and improved learning and memory performance, based on tests using a Morris water maze [117]. Most studies have focused on the pathway by which CBS catalyzes the reaction with substrate homocysteine to produce cystathionine; little attention has been paid to another pathway, in which CBS produces H_2_S from L-cysteine as a substrate. SAM enhances CBS function in both metabolic pathways which is much reduced in AD brains. A recent study found that H_2_S and SAM were reduced but that homocysteine was upregulated in AD brains [113]. These findings indicate that both H_2_S and SAM are reduced; amounts of H_2_S may be associated with the cognitive deterioration in this disease.

Furthermore, in AD, neurons are degraded via activated neuroinflammation, oxidative stress, and neuron apoptosis. Homocysteine, a pivotal risk factor for AD, has deleterious effects on cognitive function. A recent study of homocysteine-exposed rats found that H_2_S ameliorated homocysteine-imparted cognitive dysfunction; this may play a constructive role via inhibiting reactive aldehyde aggregation, conserving GSH homeostasis, and enhancing aldehyde-dehydrogenase 2 activity and expression in the hippocampus [118]. Additionally, the A*β* cascade theory is considered as a major pathogenesis that may impart AD via oxidative stress and changes in synapses [119]. Hence, H_2_S may reverse A*β*-imparted cognitive deficiency by decreasing the generation of A*β* and repressing the downregulation of CBS and 3MST [120]. Moreover, one study found that advancement of AD can be delayed by treatment with H_2_S donors or spa waters rich in H_2_S content, targeting multiple pathophysiological mechanisms. In that study, decreased TNF-*α* and B cell lymphoma 2 (Bcl-2) expression resulted in attenuation of morphological alterations in the hippocampus and improved spatial learning and memory ability [121]. In other AD models, the cytotoxic lipid oxidation product 4-hydroxynonenal was scavenged using H_2_S therapy, providing a novel hope in the fight against AD via the neuroprotective effects of H_2_S [122]. It has been shown that deficiencies in H_2_S biosynthesis are involved in AD and that exogenous H_2_S may have therapeutic potential by decreasing A*β* protein plaques.

### 7.3. HD

HD is an autosomal dominant disease associated with a mutation in the gene encoding huntingtin (Htt) following to extended polyglutamine repeats of mutant Htt (mHtt) which elicits oxidative stress, neurotoxicity, motor, and behavioral changes. HD is featured by highly selective as well as serious damage to the corpus striatum that regulates motor function [116]. In HD, as in other neurodegenerative diseases, GSH and GSH-dependent enzymes are dysregulated. Plasma samples of HD patients were found to have lower GSH contents compared to age-matched controls [123]. In addition, GPX activity in erythrocyte samples was lower in HD patients than in age-matched controls [124]. In another study, it was reported that there was no difference in GPX activity in cultured fibroblasts from HD versus non-HD patients [125]. The HD mouse model R6/2 showed an increased GSH content in mitochondria isolated from the cortex and striatum [126]. The authors demonstrated that enhancement of GSH may be a compensatory mechanism for elevated ROS production, although they did not measure ROS or other products of oxidative stress precisely.

Surprisingly, the dominant expression of CBS in the brain in a recent study revealed the importance of CSE in the manifestation of HD, an autosomal-dominant disease associated with a mutation in the gene encoding Htt [127]. Hence, HD is thought to be the result of highly selective and profound damage to the corpus striatum, which maintains motor function. This may reflect selective small G protein Rhes (gene) binding to mHtt, enhancing its neurotoxicity [128]. There is a massive aggregation of CSE, the biosynthetic enzyme for cysteine, in HD-diseased tissues, which may mediate HD pathophysiology. Defects that occur at the transcriptional level seem to reflect the influence of mHtt on specificity protein 1 and transcriptional activation of CSE as a pathogenic mechanism; supplementation with cysteine reverses abnormalities in HD tissue cultures and in intact mouse models of HD, demonstrating therapeutic potential [129]. In this study, CSE deficiency was found in brain tissues but not in the cerebellum of HD patients, in line with the relative susceptibility of these brain regions to HD (Figure 3). Additionally, in Q175 and R6/2HD murine models of HD, CSE expression is downregulated in the striatum, cortex, hippocampus, and brainstem, but not in the cerebellum. CSE-knockout mice display impaired Rota rod performance and an abnormal hindlimb clasping and clenching phenotype that is reminiscent of murine models of HD. These HD-related phenotypic changes are reversed by exogenously supplied cysteine [107]. In mice treated with an H_2_S-releasing derivative of naproxen (ATB-346), there was a marked acceleration in the recovery of lost motor function and further enhancement of anti-inflammatory effects [130].

Additionally, H_2_S stimulates various cytoprotective pathways [43]. It is not clear whether the pathophysiological influences of CSE aggregation in HD reflect its role in producing cysteine or H_2_S. It is thought that treatment with H_2_S donors will be useful in the treatment of HD [129]. The capability of CSE and cysteine to reverse oxidative stress and lethality in HD cells demonstrates that cysteine supplementation might be useful in HD treatment. Cysteine deficiency has been found in oxidative stress and aging [131]. In the brains of HD patients, CSE levels are greatly decreased in the striatum, moderately decreased in the cerebral cortex, and unchanged in the cerebellum, reflecting the relative susceptibility of these brain regions damaged by HD. A study based on a CSE model of HD demonstrated therapeutic effects of cysteine and NAC in mice with HD. That study concluded that NAC supplementation may be useful in treating diseases associated with impaired reverse transsulfuration and oxidative stress [132]. Further studies are needed to find out the exact pathways about the roles of H_2_S in HD.

### 7.4. ALS

ALS is a debilitating neurodegenerative disease that causes muscle atrophy and paralysis leading to death. ALS is the result of selective degeneration of motor neurons. Some studies have shown that astrocytes expressing a mutation in the enzyme SOD can accelerate motor neuron death [133]. While ALS is regarded as a degenerative disease of the upper and lower motor neurons, damage is not confined to motor neurons, with sensory and axonal projections also affected but to a lesser extent [134]. Various SOD mutations have been shown to result in this distinct pathology. This phenomenon has been reported in mice harboring different SOD1 mutations, such as Gly37Arg, Gly85Arg, and Gly93Ala. All three distinct mutations result in neurodegeneration [135]. As an example, Gly93Ala mice, but not Gly37Arg mice, have elevated levels of oxidized proteins related to disease progression in the spinal cord [136]. Besides the oxidative stress involved in reduced scavenging of superoxide ion, other studies reported accumulation of GSH *in vitro* to be associated with motor neuron cell death, which stimulates ALS [137]. GSH and GSH-dependent enzymes appear to be dysregulated in ALS (Figure 3). For example, in one study, erythrocyte GSH content was noticeably lower in ALS patients than in age-matched controls. Levels of H_2_S in cerebral tissue in the familial ALS (fALS) mouse model SOD1G93A showed that increased levels of H_2_S distorted H_2_S metabolism in ALS [98].

H_2_S is regarded as an essential biological gaseous transmitter at relatively low concentrations. It acts as a neuromodulator and neuroprotectant and regulates physiological functions to repress oxidative stress. In contrast, some data imply that higher concentrations of H_2_S in ALS have toxic effects. L-homocysteine is degraded during H_2_S synthesis. Moderate levels of homocysteinemia are seen in patients with spinal cord injury and ALS [138]. Others with neurological diseases, such as AD, dementia and schizophrenia patients, also show increased homocysteine levels [139]. Homocysteine imparts oxidative stresses and deoxyribonucleic acid (DNA) damage, while H_2_S has the opposite effect. It can be concluded that decreased amounts of CBS that result in dysregulation of homocysteine metabolism or H_2_S synthesis might be a vital factor in the pathogenesis of incidental and late neuronal disorders [140]. From the above discussion, it can be concluded that H_2_S is involved in the pathogenesis of ALS, as found in several studies, and it could be an important marker for diagnosis of ALS in patients.

The above discussion of these neurodegenerative diseases clearly shows that H_2_S plays a neuroprotective role by combating oxidative stresses in the CNS to protect the body.

## 8. Role of H_2_S in Neuroinflammation

Inflammatory processes have been described in many neurodegenerative diseases, including AD, PD, HD, and ALS. As neuroinflammation is considered as a key factor in neurodegeneration, many therapeutics are aimed at delaying or stopping advancement of inflammation in neurodegenerative diseases [141]. For example, lipopolysaccharide (LPS) causes neuroinflammation, neuronal ultrastructure impairment, and cognitive defects. LPS links to immune cells such as monocytes, dendritic cells, macrophages, and B cells, thereby increasing the secretion of proinflammatory cytokines, NO, and eicosanoids [142]. Treatment with NaHS decreases LPS-induced inflammation in both primary cultured microglia and immortalized murine microglial cells. It is speculated that H_2_S inhibits NO synthase and p38 MAPK signaling pathways in a concentration-dependent manner. Suppression of H_2_S generation by silencing CSE in LPS-stimulated macrophages results in enhanced generation of H_2_S [143]. Levels of proinflammatory cytokines are lower after CSE silencing. Microglia and astrocytes, regarded as the immune cells of the brain and spinal cord, are the main active immune defense of the CNS. They impart inflammatory action by inducing nuclear factor-*κ*B (NF-*κ*B), releasing the inflammatory mediators TNF-*α*, interleukin (IL-6), and nitrite ions, and downregulating CBS and H_2_S [144]. These inflammatory factors are involved in tissue repair but may also stimulate further tissue injury and cause cell death. This effect is slightly reversed in cells pretreated with NaHS, demonstrating the anti-inflammatory effects of H_2_S [40]. It is unclear whether the anti-inflammatory mechanism involves a direct effect of H_2_S on astrocytes and microglia or an indirect effect via inhibiting the release of proinflammatory factors [145].

Moreover, AMP-stimulated protein kinase (AMPK) is recognized as a central factor in inflammation [146]. One study demonstrated inhibition of neuroinflammation by activation of AMPK by H_2_S, supporting earlier findings on the inhibitory effect of activation of AMPK against inflammation. Although AMPK has been described as a therapeutic intervention in various diseases, the discovery of H_2_S-imparted AMPK activation via the calmodulin-dependent protein kinase *β* (CaMk*β*) makes H_2_S an interesting anti-inflammatory target. It can be concluded that H_2_S imparts pivotal anti-inflammatory functions, due to its interaction with inflammation-related LPS, microglia, astrocytes, and AMPK [145].

To sum up, while combating oxidative stresses, H_2_S plays a neuroinflammatory role by inhibiting the release of proinflammatory factors in the CNS.

## 9. Further Investigations

H_2_S is regarded as a ubiquitous molecule with essential roles in a wide range of physiological and pathological processes. Various H_2_S-mediated therapies have been studied as potential catalysts of this unique mediator in preclinical and early clinical testing. The goal for developing H_2_S-based therapeutics is to enhance efficiency and reduce toxicity compared with existing therapies. Ongoing studies range from simple approaches, such as the use of zero valent sulfur, to sophisticated tactics, such as targeted H_2_S release to specific organelles. Further advancement of pH, oxygen, and free radical-sensitive donors will be helpful on the way to achieving selective delivery of H_2_S. Agents that stimulate the various H_2_S-producing enzymes (CSE, CBS, and 3MST) specifically are attractive therapeutic candidates to study. However, research in the field of H_2_S is hindered by a lack of specific inhibitors of the various enzymes involved in the synthesis of this gasotransmitter. Several enzymes, such as CSE, have been identified as substantial therapeutic targets for developing potent and highly selective inhibitors for diagnostic and therapeutic applications. Greater understanding of the mechanism of H_2_S release and modulation of synthesis is required to monitor H_2_S levels *in vivo* and to improve H_2_S-based therapeutics.

## 10. Conclusions

H_2_S, a commonly known toxic gas, plays a homeostatic role in the body by acting as an antioxidant against oxidative species such as ROS and RNS. H_2_S is generated from enzymes such as CBS, CSE, CAT, and 3MST. Higher or lower amounts of H_2_S are associated with various CNS diseases including AD, PD, HD, and ALS; therefore, H_2_S level serves as a marker for detecting these diseases. Considering that H_2_S was previously regarded as a poisonous gas, it is surprising that proper amounts of H_2_S are required in the body; decreased H_2_S levels cause neurodegenerative diseases, and induction of H_2_S can ameliorate disease conditions. The proper maintenance of H_2_S via biogenesis and catabolism functions in cell-signaling pathways. H_2_S contributes as an antioxidant and as an antineuroinflammatory agent. Furthermore, H_2_S exerts protective effects in neurological systems by shielding neurons against hypoxic injury, preventing hypochlorous acid-mediated oxidative damage, enhancing GSH generation, and repressing oxidative stress in mitochondria. Further studies are required to develop H_2_S-based therapeutics to treat neuroinflammatory diseases.

## Abbreviations

AD: Alzheimer's disease
ALS: Amyotrophic lateral sclerosis
AP-5: Amino-phosphonopentanoate
AMPA: *α*-Amino-3-hydroxy-5-methyl-4-isoxazole propionic acid receptor
AMPK: AMP-stimulated protein kinase
ATP: Adenosine triphosphate
Bcl-2: Basal cell lymphoma-2
CBS: Cystathionine *β*-synthatase
CSE: Cystathionine *γ*-lyase
CAT: Cystathionine amino transferase
cAMP: Cyclic adenosine monophosphate
CNQX: 6-Cyano-7-nitroquinoxaline-2,3-dione
CNS: Central nervous system
CO: Carbon monoxide
Ca^2+^: Calcium ion
CSF: Cerebral spinal fluid
CFTR: Cystic fibrosis transmembrane conductance regulator
DMPD: N,N-Dimethyl-P-phenylenediamine
DNA: Deoxyribonucleic acid
EGF: Epidermal growth factor
EGFR: Epidermal growth factor receptor
GSH: Glutathione
GSSG: Glutathione disulfide
GCS: Glutamyl cysteine synthase
GPX1: Glutathione peroxidase
H_2_S: Hydrogen sulfide
H_2_O_2_: Hydrogen peroxide
HS^−^: Bisulfide
HCO_3_^−^: Bicarbonate ion
HD: Huntington's disease
H_2_S_n_: Polysulfide
L-NAME: L-Nitro arginine methyl ester
3MST: 3-Mercaptopyruvate sulfurtransferase
MAPKs: Mitogen-activated protein kinases
3-MP: 3-Mercaptopyruvate
mRNA: Messenger RNA
NMDA: N-Methyl-D-aspartate
NAD: Nicotinamide adenine dinucleotide
NADPH: Nicotinamide adenine dinucleotide phosphate
NaHS: Sodium hydrosulfide
NAC: Acetylcysteine
PD: Parkinson's disease
PLP: Pyridoxal-5′-phosphate
PKA: Protein kinase constant A
ROS: Reactive oxygen species
RNS: Reactive nitrogen species
SOD: Superoxide dismutase
SAM: S-Adenosylmethionine
SNAP: S-Nitroso-N-acetylpenicellamine
SNP: Sodium nitroprusside
SiRNA: Small interfering RNA
SN: Substantia nigra
TGF-*α*: Tumor growth factor-*α*.
